# Post-clear corneal phacoemulsification endophthalmitis: profile and management outcomes at a tertiary eye care center in western India

**DOI:** 10.1186/s12348-016-0115-y

**Published:** 2016-11-28

**Authors:** Aditya S. Kelkar, Jai A. Kelkar, Prajakta M. Barve, Aishwarya Mulay, Shubhangi Sharma, Winfried Amoaku

**Affiliations:** 1 National Institute of Ophthalmology, 1187/30 Off Ghole Road, Near Mahatma Phule Museum, Shivajinagar, Pune, 411005 India; 2 Division of Ophthalmology and Visual Sciences, University of Nottingham, Nottingham, NG7 2RD UK

**Keywords:** Endophthalmitis, Microbiology, Presenting visual acuity, Time of presentation, Visual outcome

## Abstract

**Background:**

Infectious endophthalmitis is a serious sight threatening intraocular inflammation that results from exogenous or endogenous spread of organisms into the eye.A retrospective case series to study the profile of endophthalmitis following clear corneal phacoemulsification in western India between years 2008 and 2014 was held in the National Institute of Ophthalmology, Pune, India. Cases of endophthalmitis post-clear corneal phacoemulsification were reviewed pertaining to demography, clinical history, surgeon experience, surgical complications, time of onset following surgery, duration between onset of symptoms and presentation to the center, presenting visual acuity and at follow-ups, slit-lamp examination and ultrasound findings, vitreous tap culture results, treatment, and final functional and anatomical outcomes.

**Results:**

Of 60 cases, 34 were operated in the tertiary center and 26 were referred. The incidence of endophthalmitis post clear corneal phacoemulsification performed at the tertiary center was 0.17%. Mean time delay between onset of symptoms and presentation to the tertiary care center was 2.6 days. Fifty percent cases were culture +ve, of which 80% were Gram +ve and 20% were Gram −ve, no fungal isolates. Coagulase –ve staphylococcus was the most common causative organism; rare isolates included *Sphingomonas paucimobilis* and *Streptococcus mitis*. Twenty-six eyes underwent primary vitrectomy. Mean presenting visual acuity was 2.14 ± 0.07 logMAR units which improved to logMAR 0.98 ± 0.12 at final follow-up. Presenting VA was >20/200 in 13.3% and <HM in 60% cases. 66.7% of eyes had visual improvement; 26.7% cases achieved VA 20/40 at final follow-up. Gram +ve and culture –ve cases, better presenting VA, and less time delay between onset and presentation had a favorable visual outcome.

**Conclusions:**

The shift of the clinico-microbiological spectrum of endophthalmitis could be due to change in surgical technique to clear corneal phacoemulsification. Predictors of good visual outcome include good presenting visual acuity, early presentation to the center, culture negativity, and coagulase negative organisms.

**Electronic supplementary material:**

The online version of this article (doi:10.1186/s12348-016-0115-y) contains supplementary material, which is available to authorized users.

## Background

Postoperative endophthalmitis is an intraocular inflammatory condition due to infection from microbial organisms (bacteria, fungi or, on rare occasions, parasites) that enter the eye during the perioperative period [1]. Endophthalmitis is potentially the most devastating complication of cataract surgery, and despite optimal management, the visual outcome is poor in many cases [2]. Early diagnosis and aggressive treatment with appropriate antimicrobial therapy, as well as surgical intervention, are mandatory for optimal visual outcomes. In India, cataract surgery continues to be the most common cause of postoperative endophthalmitis [3–6].

Several risk factors have been described as associated with post-cataract endophthalmitis, including the type of surgery, incision site, seniority of surgeon, surgical complications, and systemic factors [5–7]. A few studies have previously reported on post-cataract endophthalmitis from different parts of India as well as abroad [2–13]. These reports suggest that there may be differences in occurrence/incidence, type of surgery associated with higher predisposition, and the causative infective organisms in the different geographic areas previously studied. However, to the best of our knowledge, there are no studies from western India on the subject.

The purpose of this study was to investigate the profile of endophthalmitis following clear corneal phacoemulsification at a tertiary eye care center in western India, with emphasis on the clinical presentation, risk factors, microbiological profile, treatment outcomes, and predictors of good visual outcome.

## Methods

This was a retrospective study of all eyes that were diagnosed as having endophthalmitis following clear corneal phacoemulsification through superior or temporal incisions between January 2008 and December 2014, and with at least 3 months follow-up in a tertiary eye care center in western India. Data included patients operated at the tertiary eye care center, as well as referred patients who were operated elsewhere within the region. All cases included in the study were operated by a fully trained ophthalmologist. Data was collected by reviewing the endophthalmitis register and the electronic medical records of all cataract surgeries operated between January 2008 and December 2014. Patients who developed endophthalmitis following small incision cataract surgery (SICS), phacoemulsification through scleral tunnel, and phacoemulsification combined with filtration surgery/vitrectomy and patients with inadequate data regarding the type of cataract surgery were excluded from the analysis. This study received approval from our institutional ethics committee and complied with the tenets of the Declaration of Helsinki.

Clinical diagnosis of endophthalmitis was based on the presence of a combination of one or more of presence of hypopyon, fibrinous membrane in anterior chamber, and vitreous haze. The presence of other symptoms and signs, such as pain, photophobia, reduced vision, ciliary injection, eyelid, or corneal edema, was supportive of the diagnosis.

All patients with endophthalmitis were admitted in the first instance. The treatment was guided by Early Vitrectomy Study (EVS) [14] protocol. Vitrectomy was done immediately followed by intravitreal injection of antibiotics (vancomycin 1.0 mg/0.1 mL and ceftazidime 2.25 mg/0.1 mL) and steroids (dexamethasone 4 mg/0.1 mL) only if patients presented with vision worse than hand motions (IVAS + PPV group). When the presenting vision was hand motions or better, an initial vitreous tap/biopsy followed by intravitreal antibiotics (vancomycin 1.0 mg/0.1 mL and ceftazidime 2.25 mg/0.1 mL) and steroids (dexamethasone 4 mg/0.1 mL) was performed (IVAS group). These were repeated as clinically indicated, by non-response, usually at 48 h. Systemic antibiotics were not administered. The procedures were carried out in the operation theater under full aseptic precautions.

Samples for microbiologic evaluation were collected from the aqueous humor or vitreous humor just before intravitreal administration of antibiotics or during vitrectomy and were sent to the laboratory for microscopy for Gram staining, potassium hydroxide mount, culture, and sensitivity. One hourly topical fortified vancomycin (5%), ceftazidime (5%), prednisolone eye drops (1%), and tropicamide eye drops (0.8%) at bedtime were started and tapered gradually at 3-day intervals depending on the response to treatment. No systemic antibiotics were given. A favorable anatomical outcome was defined as the absence of any inflammation at the end of the treatment period. A favorable functional outcome was deemed if the eye achieved best-corrected visual acuity (VA) >20/200 at the final follow-up visit.

Data collected included patient demography, clinical history (including posterior capsule rupture), details of surgery including surgeon grade/seniority, surgical complications, time of onset following cataract surgery, duration between onset of symptoms and presentation to the eye care center, symptoms and signs, presenting visual acuity, and at follow-up examinations after endophthalmitis (1 week, 1 month, 3 month, 6 month, and last follow-up), slit-lamp examination findings, ultrasound B scan findings, aqueous or vitreous tap for culture and sensitivity, type of treatment, and final functional and anatomical outcomes.

Statistical analysis was performed using the statistical software SPSS, version 20.0 (Chicago, IL, USA). The association of final visual acuity with various risk factors was analyzed by using chi-square test or Fisher’s exact test (expected cell frequency < 5). Subgroup analysis of intravitreal steroids with vitrectomy group (IVAS + PPV) vs. intravitreal injection and steroids group (IVAS) was done using Student’s unpaired *t* test. Comparison between mean final visual outcomes with respect to culture positivity was done using ANOVA test. A *p* value <0.05 was considered statistically significant.

## Results

The total number of phacoemulsification surgeries performed in the tertiary center over the study period was 19,541. All these were clear corneal superior site phacoemulsification procedures, and 403 (2.06%) had posterior capsular tear (PC tear). We were unable to determine the total number of phacoemulsification procedures undertaken in each of the referral centers.

A total of 60 patients of endophthalmitis following clear corneal phacoemulsification were evaluated for the study of which 26 patients were referred from other centers. Of these, four patients (6.66%), all from the tertiary eye care center, had eventful phacoemulsification with posterior capsular rupture. In all four cases, anterior vitrectomy was performed and the IOL was placed in the sulcus. Other cases of endophthalmitis occurred in two eyes following SICS, two following phacoemulsification through scleral tunnel, and three eyes where there was inadequate data regarding the type of cataract surgery performed and were excluded from the analysis. Amongst the 60 cases, a superior corneal incision was used in 48 (80%) and a temporal corneal incision in 12 (20%) cases. All eyes with endophthalmitis following temporal incision phacoemulsification were referred from other centers. Excluding referred cases, the incidence of endophthalmitis following clear corneal phacoemulsification surgeries at our tertiary center was 0.17%. The incidence of endophthalmitis in cases complicated by a posterior capsular rent was 0.99%. The annual incidence of endophthalmitis in the tertiary center following clear corneal phacoemulsification is summarized in Table 1. The incidence of endophthalmitis for the referred cases due could not be calculated due to lack of adequate data. None of the referred cases of endophthalmitis were complicated by posterior capsular tear.

**Table 1:** Incidence of endophthalmitis 2008–2014

Year	No. of cases of endophthalmitis/total no. of cases operated at the tertiary eye care center	Incidence
2008	6/2062	0.29
2009	6/2386	0.25
2010	4/2611	0.15
2011	5/2791	0.18
2012	6/3568	0.17
2013	4/2988	0.13
2014	3/3135	0.09
Total	34/19,541	0.17

All the cases including referred patients were operated by fully trained ophthalmologists. The seniority of the operating surgeon is summarized in Table 2 and Additional file 1: Table S1 and shows that the junior surgeons had a higher occurrence of endophthalmitis for the patients operated in the tertiary center, although this difference was not statistically significant (*p* value = 0.38 by chi-square test). We were unable to determine such figures for the referred cases due to lack of adequate data. The mean duration of follow-up was 183 days (90–930 days).

**Table 2:** Seniority of surgeons—tertiary center operated cases

Surgical experience of the surgeon in years	No of cases of endophthalmitis at tertiary eye care center/no of cases operated	Incidence of endophthalmitis
>10 years	7/5180	0.13%
5–10 years	17/10,397	0.16%
1–5 years	10/3964	0.25%
Total	34/19,541	0.17%

### Demographic data

There were 32 (53.3%) males and 28 (46.7%) were females. The mean age of our patients was 72.3 years (range 58–85 years). Endophthalmitis was always unilateral and occurred in the right eye in 36 (60%) and the left eye in 24 (40%) of cases. Associated systemic risk factors included diabetes mellitus in 8 (13.3%), systemic hypertension in 14 (23.3%), diabetes and hypertension in 4 (6.7%), and others including bronchial asthma and ischemic heart disease in 4 (6.7%) of cases. No systemic risk factors were identified in 30 (50%) of cases.

### Presenting visual acuity

The mean presenting VA was 2.14 ± 0.07 logMAR units. Presenting VA was >20/200 (<1.0 logMAR) in 8 (13.33%) and 20/200 to >HM+ (logMAR 1.0–2.2) in 16 (26.7%). A subset of 36 cases (60%) presented with visual acuity of ≤hand movements (HM) (logMAR > 2.3), including 10 (16.7%) with HM (logMAR 2.3), and 26 (43.3%) who had VA of < HM (logMAR > 2.3) and therefore proceeded to vitrectomy.

### Clinical presentation

The time interval from surgery to presentation with endophthalmitis varied from 1 day to 3 months. The majority of the patients 34 (56.66%) presented within the first postoperative week, median—7 days (range 1–90 days) (Table 3).

**Table 3:** Interval from surgery to presentation with endophthalmitis

Time interval	No. of cases	% of cases
1–7 days	34	56.66
8–14 days	6	10.00
15–28 days	10	16.66
1–2 months	8	13.33
2–3 months	2	3.33
Total	60	100

The mean time delay between onset of symptoms, as determined from the history, and presentation to the eye care center was 2.6 days (range 2 h–15 days). 

Treatment was initiated within 2 h of presentation to the eye care center in all cases. This time interval between onset of symptoms and presentation to the eye care center was significant and affected the final visual outcome (*p* value < 0.001), with cases presenting earlier having better final visual outcome (Fisher’s exact test). (see Additional file 1: Table S2).

While all 60 patients (100%) presented with a chief complaint of hazy vision, 36 (60%) patients had associated complaints such as pain, redness, watering, and floaters, whereas rest 24 (40%) did not have any associated complaints. Of the 60 cases, 36 (60%) had hypopyon, and all 60 (100%) eyes had vitreous haze clinically which was confirmed on B scan ultrasonography at presentation. Ciliary injection was observed in 28 (47.7%), eyelid edema in 18 (30.0%), corneal haze in 34 (56.7%), and fibrinous pupillary membrane in 20 (33.3%) of cases.

### Microbiology

The analyses of aqueous and vitreous samples revealed positive bacterial etiology in 30 cases (50%). Fungi were not detected in any of the samples. Of the positive isolates, 24 (80%) cases were Gram-positive and 6 (20%) were Gram-negative bacteria. Coagulase negative staphylococcus was the most common causative organism. Amongst the Gram-negative cases, *Escherichia coli* was the most commonly identified. These microbiology results are summarized in Table 4.

**Table 4:** Microbiology—culture results

Gram-positive organisms 24 (80%)	Gram-negative organisms 6 (20%)
Coagulase negative Staphylococcus .8	*E. coli* 3
MRSA coagulase negative 5	Pseudomonas 1
*Staphylococcus aureus* 3	Klebsiella 1
*Streptococcus pneumoniae* 3	*Sphingomonas paucimobilis* 1
*Propionibacterium acnes* 1	
*Staphylococcus epidermidis* 3	
*Streptococcus mitis* 1	

*Streptococcus mitis* was identified in one eye with endophthalmitis of late onset (6 weeks postoperatively). The eye with *Sphingomonas paucimobilis* endophthalmitis presented on the first day postoperatively. Both cases improved to 20/200 at the final follow-up visit.

### Treatment

Intravitreal antibiotics and steroid (IVAS) was given in all patients as described previously. Of the 32 patients in the IVAS group, 24 required only one injection, 6 cases required two injections with an interval of 48 h between two consecutive injections, and 2 cases required three injections for non-resolving inflammation; in 1 case, this next injection was administered 48 h after the second injection, and in the other case, it was administered 1 week following the second injection.

A cohort of 28 patients required pars plana vitrectomy (PPV) with intravitreal antibiotics (IVAS + PPV group), 26 patients underwent primary 25 gauge pars plana core vitrectomy along with intravitreal antibiotic and steroid injection, while in two (3.3%) cases, vitrectomy was done after three intravitreal injections due to non-resolving inflammation.

Amongst the 26 cases that underwent primary vitrectomy with injection, 10 required one more intravitreal injection and 8 required two more injections, with an interval of 48 h between two consecutive injections.

### Final visual acuity and correlations

The mean VA at final follow-up was logMAR 0.98 ± 0.12. A favorable functional outcome (VA < logMAR 1.0 or 20/200) was seen in 40 (66.7%) eyes, with 26.7% cases achieving VA better than 0.3 logMAR (20/40) at final follow-up. Table 5 shows the correlation between presenting and final visual acuities. All eyes with VA > HM improved following treatment, with a final VA better than 20/200 in 83.33% of cases. In the 36 eyes that presented with VA of <HM (logMAR > 2.3), BCVA improved in 77.8% of cases, with 20/200 achieved in only 55.6% of cases.

**Table 5:** Correlation between presenting and final VA

Presenting VA subgroups	No. of cases	No. of cases with final VA better than presenting VA	No. of cases with final VA < logMAR 1 (>20/200)
≥20/200(≤1 logMAR)	8	8 (100.0%)	8 (100.0%)
<20/200-HM+ (logMAR 1.1–logMAR 2.3)	16	16 (100.0%)	12 (75.0%)
≤HM (≥logMAR 2.3)	36	28 (77.8%)	20 (55.6%)

Eyes with good presenting VA (≥20/200) had a 71.5% improvement in VA with a mean final visual outcome of logMAR 0.29 ± 0.11, while those with presenting VA of <20/200 (mean logMAR 2.32 ± 0.05) had a final VA of logMAR 1.09 ± 0.13 (Table 6).

**Table 6:** Correlation between presenting VA, final VA, and visual improvement across presenting VA subgroups

Presenting visual acuity subgroup	Mean presenting VA (logMar)	Mean final VA (logMar)	Improvement (%)
VA ≥ 20/200(≤logMAR 1) (*n* = 8)	1.00 ± 0.00	0.29 ± 0.11	71.5
VA < 20/200 (>logMAR 1) (*n* = 52)	2.32 ± 0.05	1.09 ± 0.13	55.4

Values are mean ± standard error of mean (SEM)

The difference between the mean presenting VA and final visual outcome was statistically significant (*p* value = 0.001). A total of 20 patients had VA < 20/200 at final follow-up.

**Fig. 1:**
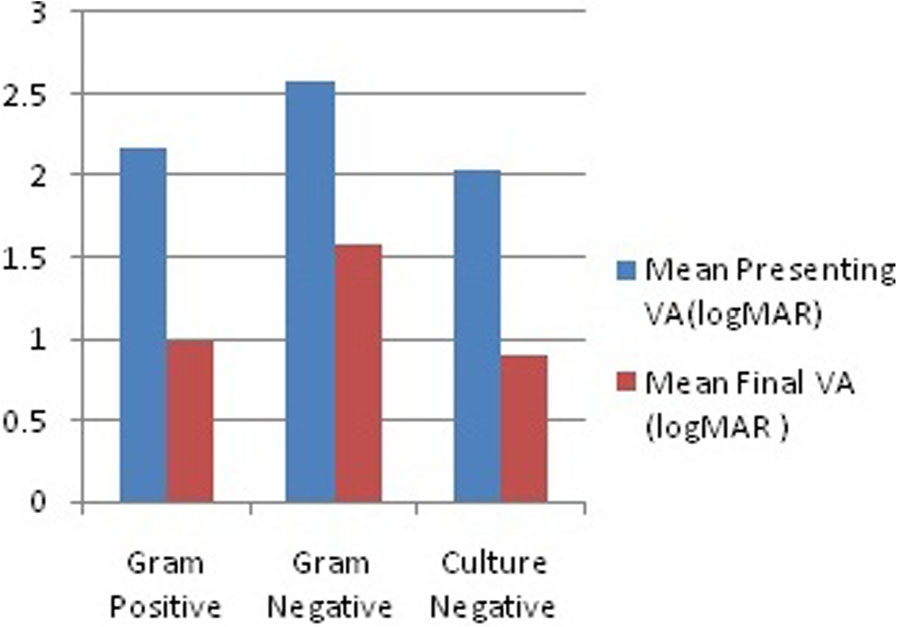
The inter-group comparison of presenting vision and final vision logMar: based on microbiology

The microbial culture-negative cases showed better mean VA at presentation (logMAR 2.03 ± 0.09). Their posttreatment outcomes were also better, with a mean final VA of logMAR 0.89 ± 0.16. The mean final visual outcome was better than 20/200 in culture-negative and Gram-positive subgroups while the mean final visual outcome was worse than 20/200 in Gram-negative subgroup. Figure 1 shows the inter-group comparison of presenting vision and final vision logMar based on microbiology. Overall, 66.7% of Gram-positive cases and 73.3% of culture-negative cases improved to 20/200 or better while only 33.33% of Gram-negative cases improved to 20/200 or better. Thus, Gram-positive and culture-negative cases seemed to have a favorable visual outcome while eyes with Gram-negative cultures had a worse final visual outcome. This difference was, however, not statistically significant (*p* value = 0.18 by ANOVA test). These are summarized in Additional file 1: Table S3.

The mean final VA was slightly better in the IVAS group (logMAR 0.89 + 0.15) versus combination of PPV + IVAS (logMAR 1.09 + 0.18) although the difference was not statistically significant (*p* value = 0.38, Student’s unpaired *t* test) (Table 7).

**Table 7:** Treatment outcomes—comparison between IVAS + PPV vs. IVAS

Parameters	Injection + vitrectomy group (IVAS + PPV) (*n* = 28)	Injection group (IVAS) (*n* = 32)	*T* value	*P* value
Presenting vision (logMAR)	2.19 ± 0.11	2.11 ± 0.09	0.540	0.591^NS^
Final vision (logMAR)	1.09 ± 0.18	0.89 ± 0.15	0.890	0.377^NS^
% Improvement	52.6%	61.9%	−1.082	0.284^NS^

Values are mean ± standard error of mean (SEM). *P* values by Student’s unpaired *t* test. *P* value < 0.05 is considered to be statistically significant

*NS* statistically non-significant

By using multivariate binary logistic regression with response variable as the final outcome of vision and with risk factors as age, time delay between onset of symptoms and presentation; day of presentation; presenting features such as corneal haze, hypopyon, flare, and cells; vitreous haze; and pupillary membrane, there is a significant association with time interval >1 day (*p* value = 0.046, odds ratio of 4.794 with 95% CI) with poor visual outcome.

Causes of poor visual outcome included retinal detachment (three eyes), non-resolving vitreous haze (three), pigment dispersion over the lens (four), epiretinal membrane (four), macular hole (one), corneal decompensation (four), and macular infarction with sclerosed vessels and optic atrophy (one). The one diagnosed with macular hole 2 months post-vitrectomy for endophthalmitis subsequently underwent vitrectomy with ILM peeling and gas tamponade. At final follow-up, this eye achieved a BCVA of 20/125 (logMAR 0.8) with closure of macular hole. Three eyes had rhegmatogenous retinal detachment following vitrectomy for endophthalmitis. Two of these eyes underwent vitrectomy with silicone oil but had a poor final visual outcome, in spite of favorable anatomical attachment of the retina. One eye had PVR with funnel retinal detachment and had no further intervention.

## Discussion

Endophthalmitis has been studied in great detail in the past, with various publications from western countries [9, 11, 12] and China [15] as well as the Indian subcontinent [3–6, 13]. The previous studies from India were mainly during the era of extracapsular cataract extraction (ECCE), and over the transition from ECCE to phacoemulsification, and were from the north and southern parts of the country. This study, to the best of our knowledge, represents an attempt to fill the void in the literature from western India on endophthalmitis following clear cornea incision phacoemulsification.

This study aimed at evaluating the profile of all cases of post-phacoemulsification endophthalmitis patients with emphasis on the clinical presentation, microbiological profile, treatment outcomes, and predictors of good visual outcome. The catchment area of this study was predominantly urban, located in the western part of India.

The reported incidence of post-cataract surgery endophthalmitis has varied, ranging from <0.05 to >0.3% [11, 12, 16–19]. The incidence of endophthalmitis after clear corneal cataract surgery in this study of 0.17% is comparable to that from other previous studies. Similarly, posterior capsule rupture shows the higher association with endophthalmitis reported in previous studies [5, 11, 12]. In our study, the male predilection and the age range of 48 to 85 years (mean, 72 years) are similar to that reported in other studies from elsewhere [13, 14, 20, 21]. Similarly, the occurrence of hypopyon as a presenting feature in 60% and corneal involvement in 10% of our cases are comparable to 50–85.7% of hypopyon [1, 13, 14, 20, 22] and 4.8–9% of corneal infiltrate previously reported elsewhere [13, 14]. Gupta et al. [13] and Kamalarajah et al. [2] had reported a presenting VA < 20/200 in the majority (92.75 and 85%, respectively) of the patients which is similar to our study (86.66%).

The EVS study [14] has reported a 69.3% culture-positive rate from biopsies/taps in post-phacoemulsification endophthalmitis. In the various Indian studies, culture positivity of endophthalmitis cases was reported to be 38–58% [4, 13, 20]. The culture-positive rate of 50% from our study is comparable to these earlier reported figures. The microbiological results from our study revealed coagulase negative Staphylococci as the most common causative organism in concordance with the world literature on endophthalmitis [23] and some Indian studies [3, 20]. However, a study of post-cataract endophthalmitis from north India [13] which included 70.5% of ECCE surgeries showed a marked microbiological variation, with 57.5% fungal cases, and *Aspergillus flavus* as the most common organism. Another study from south India [6] reported Nocardia species as the most common isolated organism, accounting for more than half of their relatively small sample size. This difference could be attributed to variations in the sample size, type of surgery, and geographical/environmental variation. Additional file 1: Table S4 gives a comparison between culture-positive rates in this study in comparison to a few other studies.

An interesting finding in our study is the identification of rare causative organisms such as *S. mitis* and *S. paucimobilis. S. mitis* acute endophthalmitis has previously been reported post-intravitreal injections and after other intraocular surgeries with devastating visual outcomes in majority of patients; however, this organism is not usually associated with post-cataract surgery endophthalmitis with late presentation [24, 25]. *S. paucimobilis*, an aerobic, Gram-negative bacillus, is another rarely reported causative organism in post-cataract surgery.

Various Indian studies have reported final visual outcomes >20/200 in 33% [13] to 54.8% [20] cases. In our study, the final VA was >20/200 in 40 (66.7%) cases. This positive difference may be explained by the earlier presentation and prompt institution of management using the EVS protocol. The difference in the causative organisms may also partly explain the better outcomes than previously reported from north and south India where fungal endophthalmitis was a more common cause of endophthalmitis [13, 20]. One of the important predictors of final visual outcome is the presenting visual acuity [21, 22]. Similar to studies reported by Gower et al. [22] and Lalwani et al. [21], the results from the present study observed that a better presenting visual acuity resulted in better final visual outcome.

Good visual outcome in our study could be attributed to prompt initiation of treatment within 2 h after diagnosis of endophthalmitis and a predominance of coagulase negative staphylococci and culture-negative cases which are generally associated with a less severe disease and hence better visual outcome [21, 26, 27]. Similar to the EVS study [14], predisposing factors for poor visual outcome in our study were poor presenting visual acuity and type of organism on culture.

Retinal/choroidal detachment, corneal edema, epiretinal membrane, vitreous opacities, neovascular glaucoma, and phthisis bulbi have previously been reported as the major causes of poor visual outcome in post-cataract endophthalmitis [21]. The causes of poor visual outcome in the present study are similar to those previously described, except that none of the eyes (in our study) developed phthisis bulbi.

Although the data was meticulously collected in this study, the retrospective non-comparative design of study, lack of polymerase chain reaction (PCR) testing in all patients are obvious limitations. However, we believe that this report from a large tertiary referral unit, which included data gathered over the course of 6 years, reflects the current scenario of endophthalmitis in western India. Polymerase chain reaction (PCR) accompanied with cloning and sequencing is one of the most sensitive, specific, and rapid molecular techniques in the detection of microbial species in clinical specimens like infectious endophthalmitis [28]. Fifty percent of patients in the current study were culture negative of which 26.7% had poor visual outcome. We feel that in these cases, PCR would have probably improved the chances of identification of organisms, which would have resulted in better outcomes in this group of patients.

## Conclusions

Post-cataract surgery endophthalmitis has wide geographical variations. In the current study, the predictors of good visual outcome in post-phacoemulsification endophthalmitis include a good presenting visual acuity, less time delay between onset of symptoms and presentation to the eye care center, culture negativity, and coagulase negative Staphylococcus as the causative organism. Urbanization, availability of modern technology for early diagnosis, and prompt management of endophthalmitis and change in surgical technique from extracapsular cataract extraction to phacoemulsification could be the reason for a shift of the clinico-microbiological spectrum of endophthalmitis, ultimately resulting in better visual outcomes.

## Additional file

Additional file 1:
**Table S1.** Seniority of surgeon- referred cases. **Table S2.** Correlation between time interval between onset of symptoms and presentation to the tertiary eye care center and visual outcomes. **Table S3.** Microbiological organism vs. visual outcomes. **Table S4.** Comparison of culture positivity and microbiologic spectrum in different studies. (DOCX 20.3 kb)

## Abbreviations

BCVA: Best-corrected visual acuity
CI: Confidence interval
ECCE: Extracapsular cataract extraction
EVS: Endophthalmitis vitrectomy study
IVAS: Intavitreal antibiotics and steroids
PC: Posterior capsular
PCR: Polymerase chain reaction
PPV: Pars plana vitrectomy
RR: Relative risk
SEM: Standard error of mean
SICS: Small incision cataract surgery
VA: Visual acuity
