# Supplementary material for: Post-clear corneal phacoemulsification endophthalmitis: profile and management outcomes at a tertiary eye care center in western India
Source: J Ophthalmic Inflamm Infect. 2016 Nov 28;6:48. doi: 10.1186/s12348-016-0115-y (PMC5126034; doi:10.1186/s12348-016-0115-y)
Supplement: Additional file 1: — Table S1. Seniority of surgeon- referred cases. Table S2. Correlation between time interval between onset of symptoms and presentation to the tertiary eye care center and visual outcomes. Table S3. Microbiological organism vs. visual outcomes. Table S4. Comparison of culture positivity and microbiologic spectrum in different studies. (DOCX 20.3 kb) [file 12348_2016_115_MOESM1_ESM.docx]

**Additional file 1: Table S1. Seniority of Surgeon- referred cases**

| Surgical experience of the surgeon in years | No of cases of endophthalmitis |
| --- | --- |
| >10 years | 6 |
| 1. – 10 years | 12 |
| 1. 5 years | 8 |
| **Total** | **26** |

| **Additional file 1: Table S2. Correlation between time interval between onset of**  **symptoms and presentation to the tertiary Eye Care Center and visual outcomes** | | | | |  |
| --- | --- | --- | --- | --- | --- |
| Time interval between onset of symptoms and presentation to the eye care center | | VA at Final follow up | | Total |  |
|  |  | VA>logMAR 1.0 | VA<logMAR 1.0 |  |  |
|  | 2 – 12 hours | 6 | 6 | 12 |  |
|  | 12- 24 hours | 0 | 18 | 18 |  |
|  | 1- 2 days | 6 | 8 | 14 |  |
|  | 3 days – 7 days | 4 | 6 | 10 |  |
|  | 7 days – 15 days | 4 | 2 | 6 |  |
| Total | | 20 | 40 | 60 |  |

By using Fisher’s exact test, p value is 0.001, hence statistically significant

**Additional file 1: Table S3. Microbiological organism vs. Visual outcomes**

| **Microbial culture outcomes** | **Mean ±SEM presenting VA** | **Mean ±SEM final VA** | **Patients with VA<logMAR 1** |
| --- | --- | --- | --- |
| Gram positive(24) | 2.17 ± 0.13 | 0.98 ± 0.17 | 16 (66.7%) |
| Gram negative(6) | 2.57 ± 0.08 | 1.57 ± 0.51 | 2 (33.3%) |
| Culture negative(30) | 2.03 ± 0.09 | 0.89 ± 0.16 | 22 (73.3%) |

**Additional file 1: Table S4. Comparison of culture positivity and microbiologic spectrum in different studies**

| **STUDY** | ***Gupta et al,2002^13^***  **(n= 124)** | ***Das et al,2011*^20^**  **(n =62)** | **EVS ^14^**  **(n= 420)** | **Present Study (n=60)** |
| --- | --- | --- | --- | --- |
| Culture +ve  Of culture +ve cases:  Gram +ve  Gram –ve  Fungal  Polymicrobial | 38%  13%  13%  57.5%  17% | 58.1%  69.4%  25%  2.7%  2.7% | 69.3%  94.2%  6.5%  0%  9.3% | 50%  80%  20%  0%  0% |
| Most Common Organism | Aspergillus flavus | Staph. epidermidis (coagulase negative staphylococcus) | Coagulase negative micrococci | Coagulase negative staphylococcus |
